# Beyond the Basics: A Novel Approach to Integrating a Social Determinants of Health Curriculum into an Emergency Medicine Course

**DOI:** 10.5811/westjem.59797

**Published:** 2023-10-11

**Authors:** Nikkole J. Turgeon, Katherine Dolbec, Florence On, Erica Lash, Emily Reed, Kateline Wallace, Adam Fortune, Katie M. Wells

**Affiliations:** * Boston Medical Center, Department of Emergency Medicine, Boston, Massachusetts; † University of Vermont, Larner College of Medicine, Burlington, Vermont; ‡ University of Vermont Medical Center, Department of Emergency Medicine, Burlington Vermont; § University of Vermont Medical Center, Burlington, Vermont

## Abstract

**Background:**

Our aim was to implement and evaluate a novel social determinants of health (SDoH) curriculum into the required fourth-year emergency medicine (EM) course at the University of Vermont Larner College of Medicine with the goal to teach students how to assess and address SDoH in clinical practice. The objectives were as follows:

1. Assess the SDoH, risk factors, and barriers to healthcare facing patients from diverse socioeconomic and cultural backgrounds in the ED.
2. Examine how social work consult services operate in the ED setting and how to identify appropriate referrals, resources, and treatment plans for patients in the ED.
3. Examine and interpret the impact health disparities have on patients in the ED and develop potential solutions to reduce these disparities to improve health outcomes.
4. Analyze the experiences and lessons learned and use them to inform future patient interactions.

**Curricular Design:**

The curriculum was developed by a workgroup that considered the following: scope; target learners; overall structure; instructional and delivery methods; and session scheduling. The curriculum consisted of four components that took place over the four-week EM course. Students completed a required end-of-course survey. Survey results underwent a mixed-methods analysis to assess student attitudes and the impact of the curriculum.

**Impact/Effectiveness:**

We received a 78.7% (74/94) completion rate for the 2021-2022 academic year. Of all respondents, 92% (68/74) indicated that they would apply lessons learned from the SDoH components of the curriculum; 74% (54/74) rated the SDoH curriculum as good, very good, or excellent; and 81% (60/74) felt that the EM course increased their understanding of diversity, equity, and inclusion as it relates to the practice of medicine. The thematic analyses revealed four main themes: 1) general comments; 2) course design; 3) interprofessional collaboration; and 4) expanding the scope of the curriculum.

**Conclusion:**

Social medicine integration into core EM courses is a generalizable approach to experiential and collaborative exposure to the social determinants of health. Of student respondents, 92% indicated they will use lessons learned from this curriculum in their future practice. This can improve the way future generations of physicians identify SDoH and address the social needs that affect their patients, thereby advancing and promoting health equity.

## BACKGROUND

Social determinants of health (SDoH) are the conditions in which people are born, grow, live, work, and age.^1^ The SDoH contribute to about 80% of a person’s overall health and underlie many health disparities that exist in different groups of people based on class, gender, and ethnicity.^2^ ^,^ ^3^ As health inequities continue to widen, the calls for teaching the SDoH and health equity to the new generations of health professionals become more urgent.^4^ ^,^ ^5^ ^,^ ^6^

Traditionally, medical education has focused primarily on the biomedical approach, which overlooks the urgent need to keep pace with the evolution of medicine’s social contract with humanity—our commitment to advocate for social, economic, educational, and political changes that ameliorate suffering and contribute to human well-being.^7^ ^,^ ^8^ ^,^ ^9^ Despite the growing obligation of medical schools to cultivate awareness and understanding of the SDoH and social inequalities, undergraduate medical education (UME) has been slow to prioritize these needs at the same level as foundational science material.^8^ ^,^ ^9^ ^,^ ^10^ ^,^ ^11^ This omission has led to the perpetuation of structural racism, transphobia, and many other forms of structural violence in medicine that exacerbate inaccessibility to basic medical care and contribute to health inequities.^12^ ^,^ ^13^ ^,^ ^14^ Moreover, the persistent health inequities in the US, especially those worsened by the COVID-19 pandemic, necessitate intentional and sustainable approaches to teaching equity-informed care. The next generation of physicians needs UME training that addresses the forces that drive health inequities such as the SDoH, social needs, and adverse structural factors such as systemic discrimination.^15^

A growing number of US medical schools have started to incorporate the SDoH into their curricula; however, many obstacles remain that prevent meaningful integration.^9^ ^,^ ^10^ ^,^ ^11^ ^,^ ^14^ These obstacles include an ideology that addressing SDoH is outside the realm of physician responsibility, limited space in the curriculum, lack of faculty knowledge, and lower prioritization due to lack of representation of the concepts on certifying examinations.^9^ ^,^ ^10^ ^,^ ^11^ Historically, pedagogical approaches in UME aimed at the SDoH have been mostly siloed into a few lecture-based sessions with few longitudinal options, and the material is often heterogeneous outside of basic definitions.^10^ ^,^ ^11^

The 24/7 accessibility of the ED and the enactment of the Emergency Medical Treatment and Labor Act in 1986, a federal law requiring that anyone coming to the ED be stabilized and treated regardless of ability to pay, solidified the role of the ED as a safety net for medical and psychosocial emergencies.^16^ As such, the ED is a unique environment to engage students in understanding and critically addressing the SDoH, as a patient’s presentation is quite often influenced by the impacts of social factors on their health.^17^ ^,^ ^18^ ^,^ ^19^ ^,^ ^20^ Currently, there is a paucity of literature on incorporating SDoH training at the UME level within emergency medicine (EM) courses.^10^ ^,^ ^11^ ^,^ ^21^

We designed a novel SDoH curriculum to address gaps and limitations of teaching SDoH that goes beyond an introductory approach and challenges students to assess SDoH and address them in clinical practice. The curriculum is incorporated into the required University of Vermont Larner College of Medicine (LCOM) fourth-year EM course, building off the foundation developed in the pre-clinical longitudinal social medicine curriculum. The pre-clinical curriculum includes the utilization of year-long, small-group discussion space for critical reflection on social medicine topics, the creation of social medicine learning objectives, cross-curricular integration with foundational sciences, and adjunctive events such as SDoH rounds.^22^ ^,^ ^23^

Our curriculum uses students’ prior knowledge of SDoH and applies that knowledge in a real-time clinical context. This allows for the contextualization and recognition of the interplay between SDoH and clinical medicine by ensuring students are encountering the SDoH in conjunction with their patients’ presenting clinical complaints rather than learning about SDoH in isolation. In this article, we explain the development of the SDoH curriculum and how we assessed its impact using a mixed-methods approach.

## OBJECTIVES

Four educational objectives were designed and mapped to LCOM competencies and programmatic objectives that are specific to LCOM and based on the standards set by the Liaison Committee on Medical Education. The core competencies at LCOM include the following: 1) patient care; 2) medical knowledge; 3) practice-based learning and improvement; 4) interpersonal and communication skills; 5) professionalism; and 6) systems-based practice^25^ (Table 1).

**Table 1.** Educational objectives of a social determinants of health curriculum and the corresponding University of Vermont Larner College of Medicine competencies each objective is linked to.

Educational objective	Corresponding LCOM competency
Assess the SDoH, risk factors, and barriers to health care facing patients/families from diverse socioeconomic and cultural backgrounds in the ED.	1-Patient care 2-Medical knowledge 4-Interpersonal and communication skills
Using ICP, examine how social work consult services operate in the ED setting and how to identify appropriate referrals, resources, and treatment plans for patients in the ED.	4-Interpersonal and communication skills 6-Systems-based practice
Examine and interpret the impact health disparities have on patients in the ED and develop potential solutions to reduce these disparities to improve health outcomes.	2-Medical knowledge 3-Practice-based learning and improvement 6-Systems-based practice
Analyze the experiences and lessons learned and use them to inform future patient interactions.	2-Medical knowledge 3-Practice-based learning

*LCOM*, Larner College of Medicine; *SdoH,* social determinants of health; *ICP*, interpersonal collaborate practice; *ED*, emergency department.

## CURRICULAR DESIGN

### SDoH Curriculum Development

The concept for the SDoH curriculum was curated using the Instructional Design Framework of Analysis, Design, Development, Implementation, and Evaluation.^32^ We first completed a literature search. We then reviewed the titles and abstracts of approximately 268 publications related to medical education, SDoH, and teaching social medicine. A singular publication was identified that specifically addressed the incorporation of a SDoH curriculum into an EM course. That curriculum takes more of an introductory approach to teaching the SDoH as opposed to our experiential and collaborative approach^21^ (Table 2).

**Table 2.** Search strategy including all search terms for literature search performed at the start of the analysis process for the social determinants of health curriculum.

Database	Strategy	Date	Number of unique publications
PubMed	“Education, medical”[mh] OR “Education, medical, undergraduate”[mh] OR “clinical clerkship”[mh] OR “schools, medical”[mh] OR “emergency medicine”[mh] OR “medical school*”[tiab] OR “medical education”[tiab] OR “medical training”[tiab] OR “emergency medicine”[tiab] OR “medical student*”[tiab] OR “clinical clerkship*”[tiab] OR “medicine clerkship*”[tiab] AND Curriculum[tiab] OR curricula*[tiab] OR curriculum[mh] AND “social determinants of health”[tiab] OR “social determinants of health”[mh]	2/2/2022	229 unique items
CINAHL	(MH “Education, Medical”) OR (MH “Education, Emergency Medical Services”) OR (MH “Schools, Medical”) OR (TI (“medical school*” OR “medical education” OR “medical training” OR “emergency medicine” OR “medical student*” OR “clinical clerkship*” OR “medicine clerkship*”)) OR (AB (“medical school*” OR “medical education” OR “medical training” OR “emergency medicine” OR “medical student*” OR “clinical clerkship*” OR “medicine clerkship*”)) AND (MH Curriculum) OR (TI (curricula* OR curriculum)) OR (AB (curricula* OR curriculum)) AND (MH “social determinants of health”) OR (TI (“social determinants of health”)) OR (AB (“social determinants of health”))	2/2/2022	24 unique items
Scopus	TITLE-ABS-KEY(((“medical school*” OR “medical education” OR “medical training” OR “emergency medicine” OR “medical student*” OR “clinical clerkship*” OR “medicine clerkship*”) W/10 (Curriculum OR curricula*)) AND (“social determinants of health”))	2/2/2022	15 unique items

Next, we designed the curriculum considering the following: scope; target learners; overall structure; instructional and delivery methods; and session scheduling. The SDoH curriculum then underwent a rigorous approval process by the LCOM Medical Curriculum Committee and was approved and implemented for the 2021–2022 academic cycle. The EM course director, the director for health equity, and two other faculty members oversaw and facilitated the components of the SDoH curriculum. The social work team played an integral role in assisting with curricular design and scheduling. Lastly, an end-of-course survey was completed. The EM course materials were provided to students through the LCOM online platform. We created a separate SDoH syllabus that detailed each of the components of the curriculum and provided a list of resources for students to use. Other materials provided were a SDoH screening questionnaire for use during the structured patient interviews and a scoring rubric for the reflection.^26^ ^,^ ^27^ ^,^ ^28^

### SDoH Curriculum Structure

The components of the SDoH curriculum were 1) a SDoH shift; 2) SDoH structured patient interviews; 3) written reflection; and 4) a solutions-focused group project.

Each student completes 14 clinical shifts over the course of four weeks. In addition to these clinical shifts, the students were assigned a four-hour SDoH shift during their first 10 days in the ED where they engaged in interprofessional collaborative practice with a social worker. The social work team is consult-driven and serves the ED and inpatient services. Our social work team volunteered to have students work with them. The social workers chose dates for the student shifts based on their availabilities, and these shifts were assigned to students at the start of the course. During the SDoH shifts, students applied their pre-clinical understanding of the SDoH along with the knowledge obtained from the social work team to help develop a plan of home care, follow-up care, or transition care dependent upon the patient’s situation.

In the first two weeks of the course, students also conducted a minimum of four structured SDoH patient interviews that were focused on understanding the patient’s needs beyond the scope of the disease process with which they were presenting. These interviews took place at the convenience of the student either during their SDoH shift with the help of the social work team, during regular clinical shifts, or on their own time. Students asked the patients questions using a questionnaire guide that we adapted from several validated screening tools.^29^ ^,^ ^30^ ^,^ ^31^ The questions involved learning about the patient’s social situation including utilization of primary care, housing insecurity, food insecurity, language barriers, bias (racial, economic, sexual orientation, etc), transportation, and alcohol or substance use.

Prior to the interview, students introduced themselves and obtained consent from the patients to discuss their social circumstances. The goal of the interview was to provide students with the time to ask questions to their patients relating to social needs. Often this was the first time students were asking these specific questions to real patients. Because we did not want time constraints to impact building rapport and trust with patients, students were not timed on the interviews. When a patient screened positive the student could either report back to social work, if this was during their SDoH shift, inform the patient’s physician of the need for a social work consult, or the student could directly place a consult through the secured social work consult email.

The second week of the course provided dedicated time for students to meet in their assigned small groups (3–5 students) to discuss patient cases identified during the SDoH shift or from their individual patient interviews. Each rotation there were approximately three to five small groups. The small group then selected one case to explore further by identifying a problem, barrier, or major health-equity issue related to the case and then developing solutions to prevent and possibly mitigate it. Students were expected to develop an action plan in collaboration with the ED faculty, residents, and social workers. Students were provided with contact information for faculty and social workers to whom they could reach out for guidance as needed. The last week of the course each small group presented to an audience of their peers, faculty, and social workers through a virtual meeting.

Finally, the individual students submitted a written reflection on their personal experience and expanded upon lessons learned, unexpected aspects involving the social side of EM, and how these concepts could be implemented into their future delivery of patient care. A scoring rubric was provided to students for reference.

## IMPACT/EFFECTIVENESS

To understand the impact on and the attitudes of the medical students toward the SDoH curriculum, we used a mixed-methods approach to analyze the end-of-course survey results. The survey questions assessing the SDoH course consisted of one “yes” or “no,” two free-response, and three Likert scale (1–5) items. There were additional questions about the overall EM course, and many students included specific comments about the SDoH curriculum when answering those questions as well. Data was collected throughout the 2021–22 academic year as each cohort of students completed the EM course. The demographics of the survey respondents consisted of fourth-year medical students at LCOM who had completed the EM course.

We tabulated quantitative responses and displayed them numerically, using percentages where appropriate. Qualitative feedback was analyzed using thematic analysis rooted in grounded theory.^24^ We selected this approach because of its compatibility with our goal of exploring individual narrative comments to understand the impact and attitudes of students toward the SDoH curriculum. Analysis of de-identified narrative comments was initially independently reviewed and iteratively coded by two authors. The authors compared codes and discussed their findings. Recurring codes were organized into categories of similar content and then further discussed and arranged into broader themes.

Coders reflected on potential biases that could influence interpretation. The first coder was a medical student at the time of analysis who completed the EM course in the 2021–22 academic year and had a scholarly interest in health disparities and curriculum development to improve the quality of care delivered to historically marginalized populations. The second coder was an attending emergency physician who was part of facilitating the EM course in the 2021–22 academic year and had an interest in improving the quality and culture of care for underserved communities.

It was not possible to individually identify any of the de-identified comments. Data management and analysis was facilitated with the use of Microsoft Excel (Microsoft Corp., Redmond, WA). The qualitative reporting was conducted in compliance with the standards for qualitative research reporting.^29^ We received 74/94 completed surveys, a 78.7% completion rate for the 2021–2022 academic year. Of all respondents, 92% (68/74) indicated that they would apply lessons learned from the SDoH components of the curriculum (Table 3).

**Table 3.** Quantitative results for the social determinants of health end-of-course survey questions.

Question/Statement	Yes	No
Will you apply lessons learned from your Health Equity Experience to your future practice?	68 (92%)	6 (8%)

	Likert scale				
	Strongly Disagree (1)	Disagree (2)	Neutral (3)	Agree (4)	Strongly Agree (5)
This course helped increase my understanding of how diversity, equity, and inclusion relate to the practice of medicine.	2 (3%)	–	12 (16%)	34 (46%)	26 (35%)
I had an opportunity to participate in the care of a variety of different patients in this course. Examples of variety include different medical conditions, diverse cultures, ethnicities, socioeconomic backgrounds, sexual orientations, and belief systems.	–	–	4 (5%)	27 (36%)	43 (58%)
	Poor (1)	Fair (2)	Good (3)	Very Good (4)	Excellent (5)
Rate the overall quality of the Health Equity Experience during your course (social determinants of health shift, small-group experience, and large-group discussion).	5 (7%)	15 (20%)	25 (34%)	16 (22%)	13 (18%)

The two narrative-response questions underwent thematic analyses that revealed a variety of themes and sub-themes. For the narrative response question, “how can we improve the health equity component of the curriculum,” we found four main themes: 1) general comments; 2) course design; 3) interprofessional collaboration; and 4) expanding the scope of the curriculum. For the narrative response question, “please comment on how this course addressed issues of diversity, equity, and inclusion (DEI),” we found two themes: 1) acknowledgment of how the SDoH curriculum addressed DEI training; and 2) awareness of how attitudes of attendings affect DEI and SDoH training (Table 4).

**Table 4.** Qualitative results of the thematic analysis performed on the results of the social determinants of health end-of-rotation narrative responses provided with exemplar quotes for each of the sub-themes.

*How can we improve the Health Equity Component of the Clerkship?*		
Theme	Sub-theme	Exemplar quotes
General comments	Positive	I thought this part was great. Much more than I’ve had in any other rotation (clinical or non-clinical) thus far in med school. I was surprised by that, but very pleasantly surprised by how much I got out of it even in a short time.
		It was the best health equity clerkship course so far.
	Negative	Remove it (SDoH curriculum), we do this during family med rotation, so it is repetitive.
	Neutral	I really thought it was great and can’t think of any improvements to be made at this time.
Course design	Structure of patient interviews	Encourage asking the SDoH questions to patients the student has already been building a relationship with. It’s so awkward going up to a random patient or asking the attending … if there are any patients with SDoH barriers.
		The questionnaire can be improved - it is very objective and the whole concept of SDoH is subjective; that extends beyond simple questions like “do you have housing/food?”
	Structure of SDoH shift	Work with social work when they are consulted when it is a patient that we saw during a normal shift so that we can better understand when social work is needed and how it is incorporated into better healthcare for our patient. It would make integrating the medicine and the social pieces more powerful and tangible.
	Reduce components	The SDoH curriculum is great and a fundamental aspect of what we should be learning as EM students. That being said, it was more work than expected, and tough during a stressful time of the year to have several added requirements. A panel where peers can talk thoughtfully about their experiences (vs a project and essay) would have been less stressful and more fulfilling.
	Variability of SDoH shift	I think shadowing the social workers is a little challenging. Often they are on the phone calling consults or are in meetings and there is little engagement for us. I think it was helpful to see all that they do and how they are integrated into patient care in the ED.
	Remove SDoH shift	I don’t think there needs to be an extra SDoH shift. I think it would be sufficient to provide students with the questionnaire and seek patients out during their shifts.
	Improve guidance for group project	I feel like we didn’t focus on solutions enough. It would have been more helpful to have longer case discussions with social work instead of shadowing them.
Interprofessional collaboration	SDoH shift was impactful	I personally thought I got more out of shadowing the social worker than the interview portion.
		The half-day with social work was awesome.
	Want more time with social work	I don’t think there needs to be an extra SDoH shift. I think it would be sufficient to provide students with the questionnaire and seek patients out during their shifts.
		Include more time for screening for SDoH and working with the social work team to provide patient care.
	Exposure to community resources	We talked about a lot of the resources that are offered at UVM when I was on my rotation, but there are so many it is easy to forget. It would be helpful to compile a list of resources that address each determinant of health to have a quick reminder of ways in which we can assist our patients if they screen positively for social determinants of health.
Expand the scope of curriculum	Want to have more of an impact	Work with social work when they are consulted when it is a patient that we saw during a normal shift, so that we can better understand when social work is needed and how it is incorporated into better healthcare for our patient. It would make integrating the medicine and the social pieces more powerful and tangible.
	Teach attendings	Make the preceptors aware of the health equity clerkship so they are on the lookout for appropriate patients and engaged in that side of learning.
		Teach doctors too.
	Exposure to mental health topics	More robust conversation about mental illness given its prevalence in the ED (especially in winter 2022 with many boarding psych patients). Eg, mental illness as a social determinant, stigma surrounding mental illness. Could also include how COVID-19 has magnified such disparities (also very apparent in the ED).
	Integrate with EM patients during regular shifts	Please allow students to complete the interviews during their time on shift. There was never a shift that was without a quiet period at some point, and patients who will screen positive aren’t always waiting for you to be done with your shift to interview them.
	Add more patient encounters	Continue to have students interview patients for SDoH.
*Please comment on how this course addressed issues of diversity, equity, and inclusion. Some examples include how the course dealt with race/ethnicity, gender, sexual orientation, religion, age, disability, political affiliation, and veteran status. Include what worked well for you along with any suggestions for improvement.*		
Theme	Sub-theme	Exemplar quotes
SDoH curriculum addressing DEI training	Impactful	Honestly awesome part of this course was getting to work with the social workers for an afternoon - I was pleasantly surprised by how much I got out of it and how much more I was thinking about SD[o]H afterwards with further patient encounters.
	Diversity of ED population	The social determinants of health shift was really beneficial in making us aware of some patients from all different walks of life. The ED is the place where we possibly see the most diverse patients, so overall it was an awesome experience.
	Increased awareness of DEI topics	This course had a full section/syllabus designated to SD[o]H that correlated with diversity, equity and inclusion in medicine. I really enjoyed the SD[o]H components and know the experiences will assist me as a future physician.
	Did not address DEI	Not a focus of this course.
Attending attitudes affect DEI and SDoH training		SD[o]H curriculum was okay but was superseded by the way some faculty speak about patients. I heard one doctor say “There is a drug addict in room XX” before saying “no way is she getting pain meds” the doctors are the biggest model for our behavior.
		I would like to see attendings encourage us to take on patients with different backgrounds and who speak languages other than English so that we can get that experience and practice. Sometimes they discourage us from seeing such patients.

*DEI*, diversity, equity, and inclusion; *SdoH*, social determinants of health; *ED*, emergency department.

Based on our results, we posit that the lessons learned through the SDoH curriculum can translate to improved patient care and health outcomes as 92% of students indicated they would apply these lessons to their future patients. We found that students are receptive to incorporating social medicine topics into standard clinical training courses and expressed desire to see further integration in the future. Students felt our SDoH curriculum could be improved by reducing the number of its components, primarily focusing on the SDoH social work shifts and the collaborative project, thus making structural changes in the course design to increase the impact for both students and patients.

The SDoH curriculum provided students with an opportunity to develop interdisciplinary skills through dedicated time to explore the role of social work for patients in the ED setting. We recognize that because social work is a consult-driven service in our hospital, this resulted in varying opportunities for students. Thus, this may have created a disparity in the experience of the SDoH shift for certain students where the focus was more on patient follow-up than consults. This is one area we intend to work on increasing the standardization for all students. Despite some variability, working with the social workers increased students’ ability to learn and understand the role of social work in the ED and how to work with them efficiently as future physicians. The SDoH shift also exposed students to some of the local community resources available to patients. These are useful skills for students to learn as they will be more adept at being able to navigate finding resources and working with social work for their future patients in new locations.

Our SDoH curriculum went further than many introductory courses because the students in the class of 2022 had completed a longitudinal social medicine course during their pre-clinical years. Students were able to apply their knowledge to develop solutions for their selected patient encounters. The small groups generated a myriad of solutions, several of which are being explored as improvements to ED systems processes. The small groups presenting their solutions elicited robust discussions with our faculty facilitators and social workers. The presentations cultivated an interest in population health, public health, advocacy and health policy, medical education, and more.

For example, because the first iteration of the curriculum occurred in the 2021–2022 academic year there were many social factors impacting patients related to the impact of the COVID-19 pandemic. One group recognized that non-domiciled patients were at risk of losing stable housing as a Vermont policy for providing hotels during COVID-19 pandemic would soon be ending.^34^ The group solution proposed was to organize meetings with members of the Legislature of the State of Vermont to advocate for the continuation of this policy. The group discussion led to additional ideas around how to navigate using media and other outlets for advocacy. Another small group also discussed housing insecurity and worked with a social work team to develop an easy-to-use flow chart for emergency clinicians to follow when discharging non-domiciled patients who were COVID-19 positive. The real-world application of the SDoH curriculum is providing an environment where students can develop skills in critical appraisal, peer-to-peer teaching, and ultimately foster lifelong learning.

Another salient point noted by students in both narrative response questions was the impact that the attitudes of physicians and other health professionals have on the significance of teaching the SDoH. This is an astute observation made by students because a 2014 qualitative study assessing the determinants of empathy during medical education found that interactions with colleagues can both promote and inhibit empathy through their role modeling of empathic and non-empathic behavior.^30^ This was one of six themes that emerged from their survey of practicing physicians. It is not hard to extrapolate that when adding in a power difference between an attending physician and a medical student, the attending physician’s role-modeling behavior will have a greater impact on the student. When those whom the students look up to are acting in opposition to what is being expected of students it can negate some of the impacts of what we are trying to accomplish.

While most of the feedback toward the SDoH curriculum was positive, we faced several challenges with the implementation of this curriculum. We encountered many of the same challenges described in the literature around incorporating social medicine topics into UME, such as differing opinions on the relevance of SDoH despite the exhaustive evidence of their impact on health equity, an already dense curriculum, and varying levels of expertise from faculty.^9^ ^,^ ^10^ ^,^ ^11^ ^,^ ^14^ ^,^ ^33^ To overcome some of the challenges both faculty and students advocated for the inclusion of the SDoH curriculum, and ultimately it was approved after rigorous review by the LCOM Medical Curriculum Committee. There was also no protected time or funding for the additional workload on the administrators, faculty, or the social work team. Faculty facilitators and the social work team volunteer their time to bring this curriculum to students. We recognize volunteering additional time of already busy faculty and social workers is not sustainable. although after years of relentless advocacy from LCOM medical students, LCOM now has a director for social medicine who is responsible for leading the efforts in addressing social medicine with LCOM medical education programs.

As with any curriculum, ours is continuously evolving. For the following academic year, we implemented changes based on student feedback. For example, students felt they did not have adequate time to meaningfully engage in each of the components of the curriculum on top of their clinical schedule. Therefore, we removed the reflection component and shortened the social work shift to three hours. As we continue to improve the curriculum, we are also working to further integrate social medicine concepts into existing lectures and sessions that have not typically made these connections in the past.

We recognize that although 92% of respondents indicated they would use lessons learned, we did not assess past a Kirkpatrick level 1 outcome.^35^ Therefore, we are continuing to improve the curriculum and our assessments by developing a delayed post-survey regarding students’ impression of how important and relevant this subject is regardless of specialty in attempts to identify any gaps in the curriculum as well as assessing pre- and post-knowledge related to SDoH before and after completion of the curriculum.^14^

There are also several limitations to be addressed for generalization to other institutions. First, our curriculum builds off the LCOM pre-clinical longitudinal social medicine curriculum, which we recognize most institutions do not have in place. This could increase the difficulty for some institutions if there is no formal SDoH teaching prior to their EM course/clerkship. However, we feel that our curriculum could easily be modified if this is the case. For example, the small-group projects could instead focus on students learning about a particular SDoH they identified instead of solutions. Second, the results for our study were subjective to each student’s experiences, and we had a small sample size of 74 respondents. Future analyses for this curriculum would benefit from the use of a validated course survey.

## CONCLUSION

Social medicine or health equity integration into core courses is a generalizable approach to experiential and collaborative exposure to the social determinants of health. We believe this is a model for a SDoH curriculum in an EM course that can be generalized to other institutions even with different baseline SDoH curricula. This represents an opportunity for UME to expand pedagogic practices beyond the specialty to include and encourage interprofessional partnerships. We found 92% of student respondents indicated they will use lessons learned from this curriculum in their future practice. And while our work is not finished, we intend to evaluate downstream impacts on the transfer of students’ SDoH knowledge to clinical care; this approach takes students further than before. This type of curriculum can improve the way future generations of physicians identify social determinants of health and address the social needs that affect their patients, thereby advancing and promoting health equity among the population.
